# Supplementary material for: Developing Folate-Conjugated miR-34a Therapeutic for Prostate Cancer: Challenges and Promises
Source: Int J Mol Sci. 2024 Feb 9;25(4):2123. doi: 10.3390/ijms25042123 (PMC10888849; doi:10.3390/ijms25042123)
Supplement: Supplementary file 1 [file ijms-25-02123-s001.zip › ijms-2767574-supplementary.pdf]

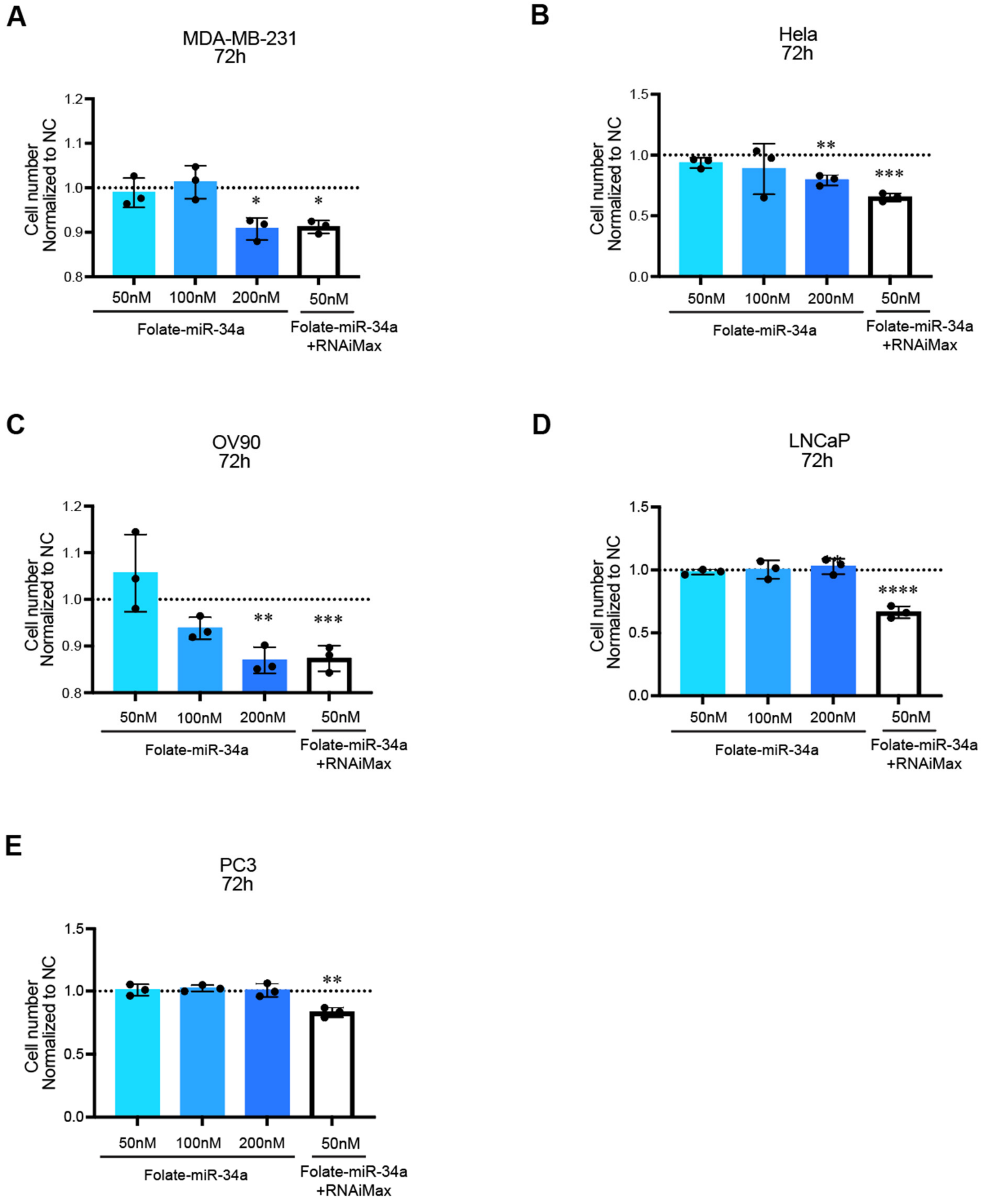


**Figure S1. Effect of folate-miR-34a on the growth of 4 cancer cell types (5 cell lines).**
MDA-MB-231 (**A**), Hela (**B**), OV90 (**C**), LNCaP (**D**) or PC3 (**E**) cells were treated with folate-miR-34a for 72 h, followed by CCK-8 assay ($n$ = 3). *, $p < 0.05$; **, $p < 0.01$; ***, $p < 0.001$; ****, $p < 0.0001$ compared to respective negative control (NC, scrambled miRNA).

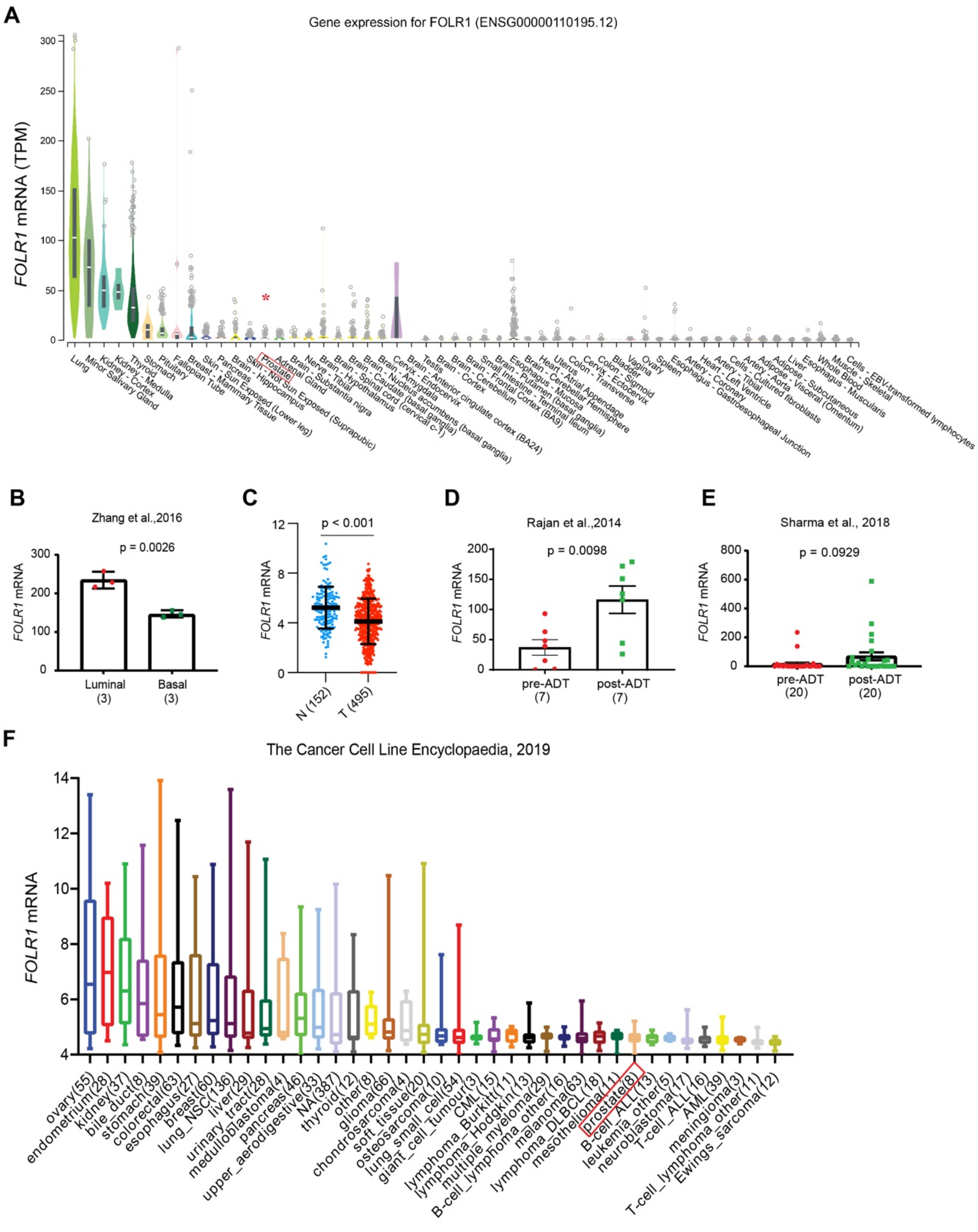


**Figure S2. *FOLR1* mRNA expression in GTEx, PCa databases, and CCLE.**

(**A**) *FOLR1* mRNA expression in normal human tissues. Data was retrieved from GTEx. Prostate is highlighted in red box and with asterisk (*).

**(B)** *FOLR1* mRNA expression in normal prostatic tissue (N) and PCa (T). Data was retrieved from GEPIA and TCGA PRAD.

(**C**) *FOLR1* mRNA expression in prostatic luminal and basal cells.

**(D- E)** *FOLR1* mRNA expression in two PCa patient cohorts that received neoadjuvant ADT.

(**F**) *FOLR1* mRNA expression in human cancer cell lines. Data was retrieved from the Cancer Cell Line Encyclopedia (CCLE). Prostate is highlighted in red box.

A

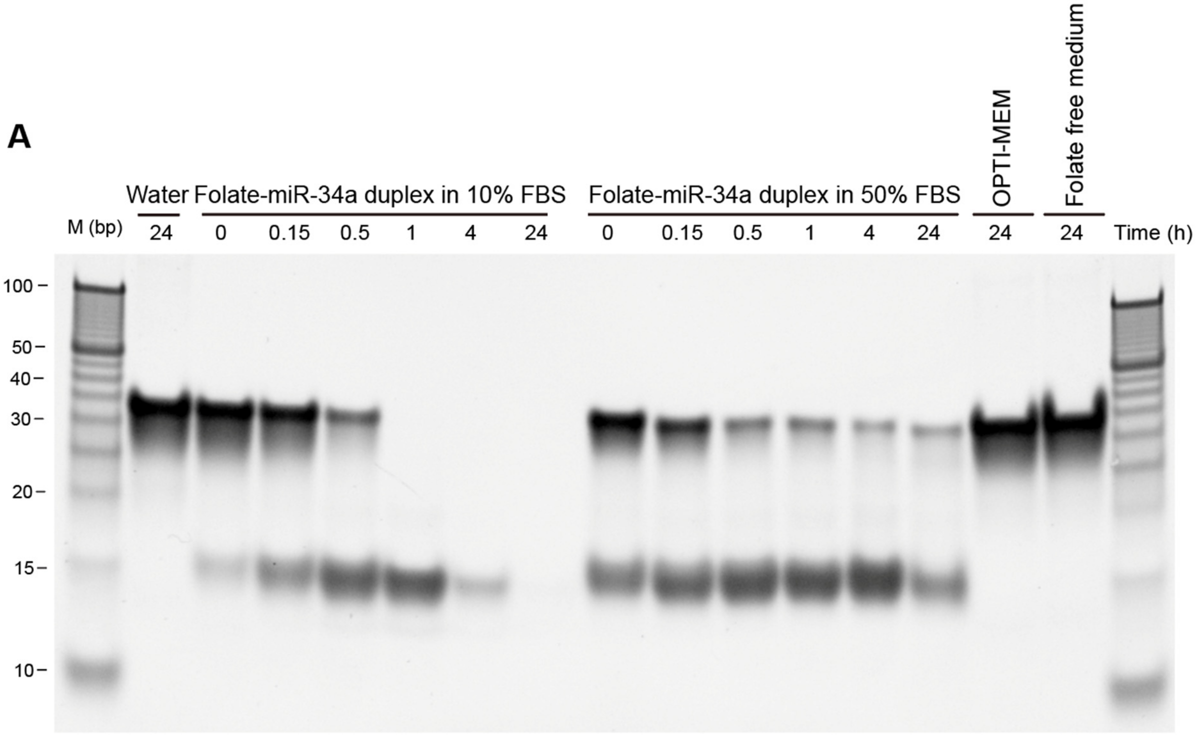


B

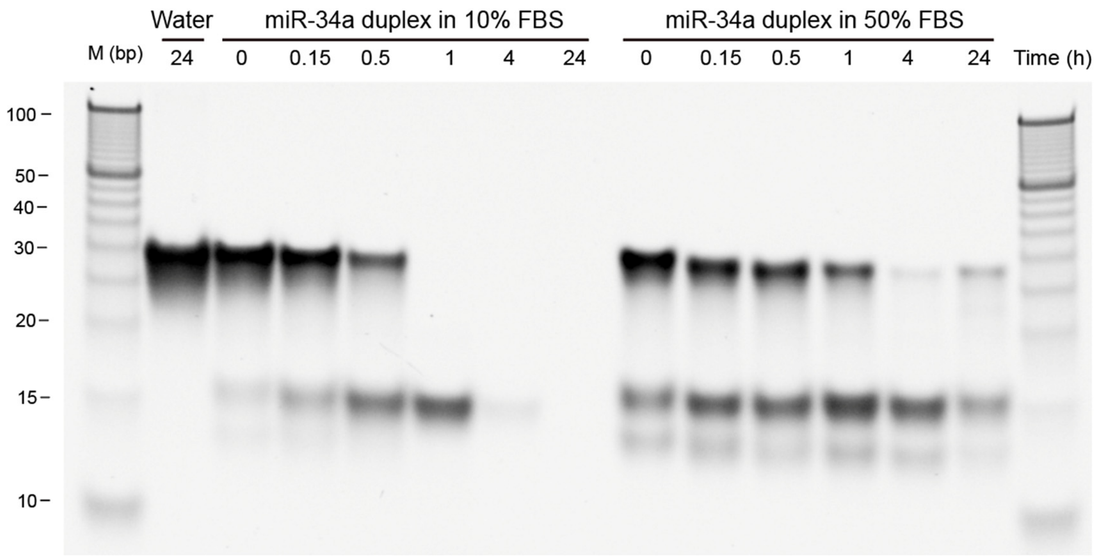


**Figure S3. Serum stability of folate-miR-34a and unmodified miR-34a.**
Representative poly-acrylamide gel images of folate-miR-34a (**A**) and unmodified miR-34a (**B**) duplexes following exposure to 10% or 50% serum over the indicated time course. Folate-miR-34a duplexes (50 pmol) or miR-34a duplexes (50 pmol) were incubated in either 10% or 50% FBS (Sigma) at 37°C for the indicated time intervals. At each time point, RNA samples were mixed with 2X RNA loading dye and stored at −20 °C. When all samples were collected at the last time point (i.e., 24 h), samples were analyzed on a 15% polyacrylamide gel in TAE followed by staining RNA using Gel Red Nucleic Acid Gel Stain (GoldBio, G-725-10).
